# Early postoperative morbidity and short-term outcomes after cytoreductive surgery and HIPEC for peritoneal surface malignancies: initial single-center experience from Palestine

**DOI:** 10.3389/fonc.2026.1885478

**Published:** 2026-09-03

**Authors:** Marwan Abufara, Qais M. Alnjoom, Amanda Siniora, Elias Yacoub, Tareq Jarrar, Ibraheem AbuAlrub

**Affiliations:** 1 Augusta Victoria Hospital, Jerusalem, Israel; 2 Palestine Polytechnic University, Hebron, Palestine; 3 St John Eye Hospital, Jerusalem, Israel; 4 Al Quds University Faculty of Medicine, Jerusalem, Palestine

**Keywords:** cytoreductive surgery, HIPEC, outcomes, peritoneal carcinomatosis, resource-limited setting

## Abstract

**Background:**

Cytoreductive surgery (CRS) with hyperthermic intraperitoneal chemotherapy (HIPEC) is an established treatment option for selected patients with peritoneal surface malignancies. However, data from newly introduced programs in resource-constrained settings are limited. We report our initial experience in Palestine, a single center, in evaluating the feasibility, perioperative safety and short-term oncologic outcomes of CRS-HIPEC.

**Methods:**

We conducted a retrospective descriptive study of all consecutive patients who underwent CRS-HIPEC at Augusta Victoria Hospital, Jerusalem, from the beginning of the program until the end of 2025. Demographic, clinicopathological, operative and postoperative data were obtained from medical records. The results Completeness of cytoreduction, postoperative morbidity, length of hospital stay, recurrence, disease-free survival (DFS), overall survival (OS), quality of life and functional status were the outcomes. Descriptive statistics were used.

**Results:**

Seventeen patients underwent CRS-HIPEC, with a mean age of 48.0 ± 13.3 years; 64.7% were female. Colorectal (58.8%), ovarian (23.5%), and appendiceal (17.6%) malignancies were the predominant primary tumors. Complete cytoreduction (CC-0) was achieved in all patients, with a mean peritoneal cancer index of 11.6 ± 10.7. The mean operative time was 430 minutes, and the estimated blood loss volume was 340 mL. Most postoperative complications were minor (91.7% Clavien–Dindo grade I–II), whereas one patient (5.9%) developed an anastomotic leak requiring reoperation. There was no 30-day mortality. The mean hospital stay was 12.7 ± 6.6 days. After a mean follow-up of 1.35 ± 0.6 years, the estimated 12-month OS was 91.7%, tumor recurrence occurred in 35.3% of patients, and the mean DFS was 14.8 ± 6.1 months. Most patients remained functionally independent (75.0%), with good or excellent quality of life (76.9%).

**Conclusion:**

The first CRS-HIPEC program in Palestine showed that this complex treatment can be safely performed in a resource-limited setting with careful patient selection and multidisciplinary collaboration. Acceptable perioperative morbidity and promising short-term results support the feasibility of program implementation, but larger prospective studies with longer follow-up periods are needed to define long-term oncologic efficacy.

## Introduction

Peritoneal carcinomatosis represents a clinically challenging pattern of cancer dissemination characterized by transcoelomic spread rather than hematogenous or lymphatic metastasis. It occurs through a distinct mechanism in which tumor cells shed from the primary tumor, disseminate within the peritoneal fluid, and implant on serosal surfaces, resulting in extensive microscopic disease that is poorly penetrated by systemic chemotherapy alone due to the peritoneal–plasma barrier (1, 2). As a result, peritoneal metastases have historically been associated with poor prognosis and are traditionally managed with palliative intent (1).

Cytoreductive surgery (CRS) in combination with hyperthermic intraperitoneal chemotherapy (HIPEC) has become an established therapeutic option for carefully selected patients with peritoneal surface malignancies (3). CRS consists of extensive peritonectomy procedures and resection of all visible tumor deposits, whereas HIPEC is administered intraoperatively with the aim of eliminating microscopic residual disease (3).

The use of HIPEC is supported by both pharmacokinetic and biological principles. Compared with systemic chemotherapy, intraperitoneal administration permits substantially higher local drug concentrations while limiting systemic toxicity (2, 4). In addition, hyperthermia generally maintained at temperatures between 41 and 43 °C enhances the cytotoxic effect of chemotherapy through multiple mechanisms, including increased tissue perfusion, improved cell membrane permeability, inhibition of DNA repair processes, and direct thermal injury to tumor cells (4, 5). Collectively, these mechanisms provide the theoretical basis for the application of HIPEC in malignancies confined to the peritoneal cavity (5).

Over the past two decades, CRS and HIPEC have increasingly been adopted for the treatment of peritoneal metastases arising from colorectal, ovarian, gastric, and appendiceal tumors, as well as malignant peritoneal mesothelioma (3). Several randomized trials and retrospective studies have reported improvements in overall and disease-free survival in carefully selected patients compared with systemic therapy alone (6, 7). However, outcomes vary widely, underscoring heterogeneity in patient populations, tumor biology, disease burden, and institutional protocols (3, 6).

Several factors have been identified as influencing outcomes following CRS and HIPEC. The extent of peritoneal involvement, evaluated intraoperatively via the peritoneal cancer index (PCI), is a key determinant of both respectability and long-term survival (7). In addition, the completeness of the cytoreduction (CC) score has repeatedly been shown to be among the strongest predictors of survival, with complete or near-complete cytoreduction (CC-0/1) required to achieve meaningful oncologic benefit (8). Patient-related factors, including baseline characteristics and performance status; nutritional indicators, such as serum albumin, and tumor-specific features, including histology, grade, and molecular characteristics, also play important roles in postoperative outcomes and overall survival (6).

Despite these developments, CRS and HIPEC continue to represent extraordinarily complex and resource-demanding interventions and are associated with significant perioperative morbidity and a prolonged postoperative course (6). Major complication rates have been reported in approximately 20–40% of patients, while perioperative mortality remains between 2% and 5%, even at high-volume, experienced centers (6). In addition, substantial variation persists among HIPEC protocols with respect to the choice of chemotherapeutic agent, dosing strategy, perfusion time, temperature parameters, and methods of administration, leading to heterogeneity in reported outcomes and ongoing debate regarding optimal practices (9).

Recent randomized studies have highlighted the need for cautious interpretation of outcomes related to HIPEC. Although CRS has consistently been associated with a survival advantage in carefully selected patients with peritoneal metastases, the incremental benefit of adding HIPEC appears to vary according to tumor type and treatment protocol, with particular uncertainty in colorectal cancer (10). These observations emphasize the importance of institutional experience, standardized protocols, and rigorous patient selection in the development and implementation of CRS and HIPEC programs (10).

Despite the global expansion of CRS and HIPEC, data from low- and middle-income countries and from newly established centers remain limited. Early reports from such settings are essential to assess procedural feasibility, perioperative safety, and early oncologic outcomes, as well as to explore how this complex therapeutic approach can be effectively integrated within resource-constrained healthcare systems.

Augusta Victoria Hospital in Palestine represents the first center in the country to initiate CRS and HIPEC programs. In this study, we present our single-center retrospective experience with CRS and HIPEC in 17 patients. Our analysis focused on baseline demographic and clinical characteristics; tumor-related factors; operative variables, including the PCI and CC score; HIPEC-specific parameters; and postoperative outcomes. By sharing our initial experience, we aim to provide regional data to the literature and to evaluate the feasibility, safety, and early outcomes of CRS and HIPEC in this emerging clinical environment.

## Methodology

### Study design

Our study was designed as a descriptive retrospective observational study reporting early experience with cytoreductive surgery (CRS) combined with hyperthermic intraperitoneal chemotherapy (HIPEC) at Augusta Victoria Hospital (AVH), Jerusalem. AVH is currently the sole provider of such services to Palestinian patients, making this cohort nationally representative.

The study included patients treated over a period of almost three years since the introduction of this service at AVH.

### Study population

All patients considered for CRS-HIPEC were reviewed by a multidisciplinary tumor board comprising surgical oncology, medical oncology, pathology, radiology, and anesthesiology specialists. Careful patient selection through multidisciplinary consensus, taking into account disease extent, resectability, expected benefit, and perioperative fitness, was a key component of implementing the CRS-HIPEC program in our resource-limited setting.

Patients were considered eligible for CRS and HIPEC if they met the following criteria:

- A satisfactory performance status, typically an Eastern Cooperative Oncology Group (ECOG) score of 0–2, indicates fitness for major surgery.
- Localized peritoneal metastases, either primary or recurrent, predominantly from gastrointestinal (colorectal, appendiceal) or ovarian malignancies.
- The absence of distant metastases on preoperative imaging, including contrast-enhanced CT of the chest, abdomen, and pelvis (CAP CT) and PET scans when available.
- Respectable disease was judged radiologically, with tumors amenable to complete cytoreduction (CC‑0/CC‑1) on the basis of the extent and distribution of peritoneal deposits.

Patients were excluded from CRS-HIPEC if they had poor performance status (ECOG), unresectable disease, or evidence of extraperitoneal metastatic spread. Diagnostic laparoscopy was not routinely performed, and decisions were made by multidisciplinary review and radiological assessment.

This study included all consecutive patients who underwent CRS and HIPEC during the study period, and no eligible patients were excluded from the analysis. A total of 17 patients received this service.

### Data collection

Patient clinical data, including surgical and anesthesia records, pathology reports, oncology notes, and inpatient clinical progress, were collected retrospectively from medical records (electronic and physical).

The collected variables included the following:

- Baseline demographic and clinical profile: Age, sex, BMI, comorbidities, smoking status, family history, Eastern Cooperative Oncology Group (ECOG) score, performance status, and serum albumin (11).
- Tumor characteristics (primary tumor origin, histology, tumor grade, recurrence, metastatic disease, lymphovascular involvement, molecular and genetic status if available and tumor burden).
- Operative details (peritoneal cancer index (PCI), completeness of the cytoreduction CC, surgical margins, operative time, estimated blood loss and intra- or postoperative complications).
- Variables related to HIPEC (chemotherapeutic agents used, duration, number of cycles, and chemotherapy toxicity).

### Operational definitions

To ensure clarity, reproducibility, and standardization, the following operational definitions were applied on the basis of retrospective chart reviews and aligned with the collected dataset:

1. Cytoreductive surgery (CRS): CRS involves the debulking of macroscopic peritoneal tumor deposits via procedures such as peritonectomy, omentectomy, organ resections (e.g., bowel, spleen, gallbladder, appendix), and adhesiolysis. Completeness was assessed with the CC score intraoperatively:

- CC-0: No visible residual disease.
- CC-1: Residual nodules ≤2.5 mm.
- CC-2: Residual nodules >2.5 mm but ≤2.5 cm.
- CC-3: Residual nodules >2.5 cm or a confluent unresectable tumor.
- A successful CRS was defined as CC-0 or CC-1 (12).

2. Hyperthermic intraperitoneal chemotherapy (HIPEC): HIPEC involves the intraoperative perfusion of heated chemotherapeutic agents into the peritoneal cavity after CRS is complete to reduce microscopic disease (13).

Important parameters:

- Temperature: 41–43 °C (maintained via inflow/outflow monitoring).
- Duration: 90 minutes in total (either one continuous cycle or two 45-minute cycles).
- Agents: Mitomycin C or cisplatin with doxorubicin.
- Cycles: 1 or 2.
- Toxicity was graded via Common Terminology Criteria for Adverse Events (CTCAE) v5.0. National Cancer Institute. (2017). Common Terminology Criteria for Adverse Events (CTCAE) (Version 5.0). U.S. Department of Health and Human Services, National Institutes of Health (14–18).

3. Peritoneal Malignancy:

- Tumor burden was measured by the Peritoneal Carcinomatosis Index (PCI) (0–39 across 13 abdominal regions (12)):
- Low: PCI 0–10.
- Moderate: PCI 10–20.
- High: PCI 20–30.
- Very High: PCI >30.
- Histological grade: 1 (well differentiated), 2 (moderately differentiated), and 3 (poorly differentiated). Definitions were aligned with standard oncology grading systems used by the National Cancer Institute (19).
- Preoperative tumor recurrence: whether the current tumor represents recurrent disease, yes: indicates prior treatment/surgery for the malignancy or does not indicate the primary presentation and first surgical intervention.

4. Outcome measures:

- Overall survival (OS): Time (months) from CRS/HIPEC to death or last follow-up.
- Disease-free survival (DFS): Time (months) from CRS/HIPEC to recurrence or last follow-up.
- Postoperative Recurrence: Indicates evidence of disease return post-CRS/HIPEC during follow-up (yes: confirmed by imaging or biopsy; categorized as local, peritoneal, or distant; no: no recurrence at last follow-up).
- Postoperative Complications: Graded by Clavien–Dindo (I–V) (20).
- Morbidity: Postoperative complications for each patient were graded according to the Clavien–Dindo classification system. Categorized as low; minor complications (Clavien–Dindo grades I–II), moderate (grade III), or high (grades IV–V) (20).
- Hospital length of stay (LOS): This is measured in days from patient admission to discharge.
- Quality of Life: Assessed via FACT-G scores where available (excellent: >100; good: 80–100; moderate: 60–80) (21).
- Functional Status: Independent, assisted, or dependent post-surgery.

### Surgical and HIPEC techniques

The aim of all patients who underwent cytoreductive surgery was complete macroscopic tumor removal. Therefore, the completeness of CC cytoreduction was documented intraoperatively.

CRS procedures are frequently extensive and involve multiorgan resections according to disease extent and intraoperative findings. Commonly performed procedures include omentectomy, appendectomy, cholecystectomy, bowel resection, peritonectomy, splenectomy, hysterectomy with bilateral salpingo-oophorectomy, diaphragmatic resection, and ureteric resection with reconstruction when needed.

Afterward, chemotherapeutic agents were prepared according to hospital practices and standards and administered in accordance with institutional protocols. HIPEC was administered via the closed-abdomen technique. The intraperitoneal perfusion temperature target was maintained at 41–43 °C with constant inflow and outflow monitoring. The total duration of perfusion was 90 minutes.

For patients with colorectal and appendiceal malignancies, mitomycin C was administered intraperitoneally at a dose of 15 mg/m2 followed by an additional dose of 5 mg/m2 during perfusion.

For patients with ovarian cancer and peritoneal mesothelioma, cisplatin (25 mg/m2) and doxorubicin (15 mg/m2) were administered intraperitoneally over 90 minutes. Intravenous sodium thiosulfate was administered at the same time for nephroprotection in patients on cisplatin-based HIPEC.

### Missing data

Data were obtained from electronic and physical medical records and operative notes. No patients were excluded because of incomplete records. Missing data were reviewed during data collection.

### Postoperative outcomes and complications

Early postoperative outcomes were documented and assessed through patient inpatient progress records. All patients were admitted postoperatively to the ICU for protective isolation and close follow-up per standard. However, extended ICU admissions were only required when clinically indicated.

Any postoperative complications were documented and later categorized via the Clavien–Dindo classification system.

More outcomes, such as post-operative infections, anastomotic leaks, the need for reoperation, the need for blood transfusion, the length of hospital stay, and the time to return of bowel function, were assessed.

### Follow-up and survival assessment

Patients were followed from the date of CRS HIPEC until the last available clinical assessment or study closure at the end of 2025. Follow-up data were collected through clinic visits at our hospital and review of electronic medical records. Structured telephone interviews were conducted to obtain updated clinical, survival recurrence, and quality-of-life information when clinic follow-up was not feasible due to the special geological situation affecting access.

The variables assessed included survival status, disease recurrence (local, peritoneal or distant), the need for additional surgical interventions, functional status and morbidity, and quality of life.

Overall survival was calculated from the date of surgery to the date of last follow-up or death. Disease-free survival (DFS) was calculated from the time of surgery to the last documented follow-up or disease recurrence. Importantly, given the fairly recent introduction of the service and complex geopolitical situation, long-term follow-up was not possible for all patients, and several patients remained in early follow-up at the time of analysis.

At the time of analysis, 14 of the 17 patients had completed at least 12 months of follow-up.

### Statistical analysis

Data analysis was performed via descriptive statistics only, given the small cohort size and exploratory nature of the study.

All the continuous variables were tested for normality with the Shapiro–Wilk test, which revealed that all the variables were normally distributed except for restoring bowel function. All continuous variables described in the present study are presented as the means ± standard deviations, with the exception of restoring bowel function, whose descriptive values are presented as medians (Q1-Q3).

Categorical variables are presented as frequencies and percentages.

No inferential or comparative statistical analyses were conducted.

### Handling of censored data

Survival outcomes were assessed from the date of CRS HIPEC until recurrence, death, last available follow-up, or study closure at the end of 2025. At the time of analysis, one patient had died, while the remaining patients were alive with varying durations of follow-up.

### Ethical considerations

The study was conducted in accordance with the ethical principles of the Declaration of Helsinki. Ethical approval was obtained from the Al-Quds university institutional review board before data collection (Ref No: 666/REC/2026). Participation was voluntary, and written informed consent was obtained from all patients. The patients were informed and explained the study objectives, eligibility criteria, voluntary nature of participation, confidentiality procedures, and the right to withdraw before submission. No personal identifiers, such as names or national identification numbers, were collected. Data were stored securely and accessed only by the research team. The results were analyzed and reported in aggregate form to protect patients’ confidentiality.

## Results

### Our experience in numbers

#### Patient characteristics

Seventeen patients received CRS and HIPEC within the study period. The average age was 48.00 ± 13.3 years, and most of them were females (64.7, n=11). 52.9% of patients had comorbidities, whereas 29.4 were current smokers. 41.2% and 29.4% reported having a positive family history of malignancy and had already had previous surgery prior to CRS. The functional status of the majority of patients was good (76.5% ECOG score 0). The average BMI was 29.2 ± 4.5 kg/m2, and the average serum albumin was 4.1 ± 0.4 g/dl (**Table 1**).

**Table 1:** Patient characteristics and background variable among study participants N=17.

Characteristics	Category	Frequency (percentage)
Age (mean ±SD)		48±13.3
Gender	Male	6 (35.3)
	Female	11 (64.7)
Comorbidities	Yes	9 (52.9)
	No	8 (47.1)
Smoking	Yes	5 (29.4)
	No	12 (70.6)
Family history	Yes	7 (41.2)
	No	10 (58.8)
Previous surgery before cytoreductive surgery	Yes	5 (29.4)
	No	12 (70.6)
ECOG Performance Status	0	13 (76.5)
	1	3 (17.6)
	2	1 (5.9)
BMI (mean ±SD)		29.2 ±4.5
Albumin (mean ±SD)		4.1 ±0.4

#### Tumor characteristics

Colorectal cancer (58.8%), ovarian (23.5%) and appendiceal (17.6%) tumors were the most common sources of primary tumors. Recurrent disease was found in 64.7% of the patients. The histologic type was adenocarcinoma (76.5%). 47.1% of the participants had metastatic disease, and 47.1% and 58.8% had lymphatic and vascular involvement, respectively. The proportion of patients with a low to moderate tumor burden (76.5%), and 23.6% of the patients had a high or very high tumor burden. Grade II was found in 47.1% of the patients, and Grade III was found in 35.3% of the patients (**Table 2**).

**Table 2:** Tumor histological, grading and molecular characteristics among study participants N=17 .

Characteristics	Category	Frequency (percentage)
Tumor origin	Colorectal	10 (58.8)
	Ovarian	4 (23.5)
	Appendiceal	3 (17.6)
Reoccurrence tumor	Yes	11 (64.7)
	No	6 (35.3)
Histology	Adenocarcinoma	13 (76.5)
	Other types	4 (23.5)
Metastatic disease	Yes	8 (47.1)
	No	9 (52.9)
Lymphatic involvement	Yes	8 (47.1)
	No	9 (52.9)
Blood vessels involvement	Yes	10 (58.8)
	No	7 (41.2)
Genetics *11 patients didn’t undergo tumor genetics study*	Wild type	6 (100)
Molecular subtype *12 patients didn’t undergo tumor genetics study*	Intact wild type	5 (100)
Tumor burden	Low	8 (47.1)
	Moderate	5 (29.4)
	High	2 (11.8)
	Very high	2 (11.8)
Histology grade	I	3 (17.6)
	II	8 (47.1)
	III	6 (35.3)

#### Surgical and HIPEC characteristics

A reduction of no residual disease or complete cytoreduction was obtained in 100% of the patients (95% CI (80.5%-100%)). Surgical complications were not observed in 13 patients (76.5%, 95% CI 50.1%-93.2%). HIPEC was performed with 76.5% mitomycin and 23.5% cisplatin combined with doxorubicin. Each patient underwent 90 minutes of HIPEC cycles, and 76.5% of them received one cycle. The mean peritoneal cancer index (PCI) was 11.6 ± 10.7. The average operating time was 430 minutes, with an average estimated blood loss of 340 mL. The toxicity of chemotherapy was not noted. In 93.8 patients, the surgical margins were negative (**Table 3**).

**Table 3:** Surgery characteristics of the among study participants N=17 .

Characteristics	Category	Frequency (percentage)
Completeness of cytoreductive surgery	No visible cancer left	17 (100) 95% CI (80.5%-100%)
Chemotherapy	Mitomycin	13 (76.5)
	Cisplatin + Doxorubicin	4 (23.5)
Chemotherapy toxicity	Asymptomatic	17 (100)
Surgical complication	Yes	4 (23.5)
	No	13 (76.5) 95% CI (50.1%-93.2%)
Margin *one patient missing*	Negative	15 (93.8)
	Positive	1 (6.3)
PCI (mean ±SD) *one patient missing*		11.6±10.7
Number of HIPEC cycles	1	13 (76.5)
	2	4 (23.5)
Duration of HIPEC cycle	90 minutes	17 (100)
Surgery duration (mean ±SD)in minutes		430 ±127
Blood loss (mean ±SD) in mL		340±201

#### Postoperative outcomes and complications

All participants were admitted to the ICU protectively, but only 2 patients had to be admitted to the ICU. 71.6% of postoperative complications were low-grade, and 91.7% of them were low-grade (Clavien–Dindo grades I--II). One patient (5.9) had an anastomotic leak that necessitated reoperation. Postoperative infections were reported in 29.4 patients, including superficial (11.8) and deep (17.6) infections. 8 patients required blood transfusions (mostly one or two units). The mean hospital stay was 12.7 ± 6.6 days, and bowel function was restored after a median of 5 (4,5) days (**Table 4**).

**Table 4:** Post operative complication among study participants N=17 .

Characteristics	Category	Frequency (percentage)
Need ICU admission	Yes	2 (11.8)
	No	15 (88.2)
Pain management	Multimodal	17 (100)
Postoperative infection	No	12 (70.6) 95% CI (80.5%-100%)
	Superficial	2 (11.8)
	Deep	3 (17.6)
Anastomosis leak	Yes	1 (5.9)
	No	16 (94.1)
Need reoperation	Yes	1 (5.9)
	No	16 (94.1) 95% CI (71.3%-99%)
Post operative complication	Yes	12 (70.6)
	No	5 (29.4)
Post operative complication grade *five patient are missing*	1	6 (50)
	2	5 (41.7)
	3	1 (8.3)
Blood transfusion needs	Yes	8 (47.1)
	No	9 (52.9)
Number of blood units	1	3 (37.5)
	2	2 (25)
	3	2 (25)
	4	1 (12.5)
Hospital stay (mean ±SD) in days		12.7±6.6
Bowel function restoring median (Q1-Q3 ) in days		5 (4,5)

#### Follow-up and survival outcomes

The mean follow-up duration was 1.35 years, with a standard deviation of 0.6. The 3- and 6-month survival rates were 100, with 95% confidence intervals of 71.3%-99% and 76.8%-100%, respectively. Moreover, the 12-month survival rate was 91.7%, with a 95% confidence interval of 73.5%-100%. Recurrence of tumors was noted in 35.3 patients, with the greatest occurrence being local tumors in 3 patients and peritoneal tumors in 2 patients, while distant metastasis occurred in 1 patient. During the follow-up, 4 patients needed further surgical operations. The majority of patients had low morbidity (64.7%), good and excellent quality of life (76.9) and were functionally independent (75). The average disease-free survival was 14.8 months, with a standard deviation of 6.1 months (**Table 5**).

**Table 5:** Follow up and survival characteristics among study participants N=17 .

Characteristics	Category	Frequency (percentage)
Follow up duration (mean ±SD) in years		1.3 ±.6
3 months survival *three patient are lost to follow up*	Alive	14 (100) 95% CI (71.3%-99%)
	Dead	0 (0)
6 months survival *five patient are lost to follow up*	Alive	12 (100) 95% CI (76.8%-100%)
	Dead	0 (0)
12 months survival *five patient are lost to follow up*	Alive	11 (91.7) 95% CI (73.5%-100%)
	Dead	1 (8.3)
Reoccurrence of tumor	Yes	6 (35.3)
	No	11 (64.7) 95% CI (61.5%-99.8%)
Reoccurrence type *among the 6 patients with reoccurrence*	Local	3 (50) 95% CI (38.3%-85.8%)
	Peritoneal	2 (33.3)
	Distance metastasis	1 (16.7)
Need other surgery during follow up	Yes	4 (23.5)
	No	13 (76.5)
Morbidity	Low	11 (64.7)
	Moderate	2 (11.8)
	High	4 (23.5)
Quality of life *four patient are missing*	Good	3 (23.1)
	Moderate	3 (23.1)
	Excellent	7 (53.8)
Functional status *one patient is missing*	Independent	12 (75)
	Assisted	3 (18.8)
	Dependent	1 (6.3)
Disease-Free Survival (DFS) in months (mean ±SD)		14.8±6.1

## Discussion

Peritoneal metastasis represents an advanced stage of abdominal malignancies and is associated with poor outcomes when patients are treated with systemic chemotherapy alone (22). CRS-HIPEC has emerged as an effective treatment strategy for select patients with peritoneal surface malignancies, particularly those originating from colorectal, ovarian, and appendiceal tumors (23). The present study describes the first single-center experience with CRS-HIPEC in Palestine and demonstrates that this treatment can be implemented safely in a resource-limited setting with appropriate patient selection and multidisciplinary collaboration.

The mean age of our cohort (48 ± 13.3 years) was lower than that reported in Western registries such as the Spanish REGECOP registry and the PSOGI PMP registry (24). Performance status remains an important determinant of tolerance to CRS-HIPEC, and most patients in our series had excellent functional status, with 76.5% classified as ECOG 0. Despite the presence of comorbidities and previous oncologic surgery in a proportion of patients, the procedure was performed safely following careful multidisciplinary assessment and a strict “fit-for-surgery” selection strategy. This approach is considered fundamental when new CRS-HIPEC programs are established and has been emphasized in previous institutional reports (22, 25).

Colorectal tumors represented the most common primary malignancy in our cohort (58.8%), followed by ovarian (23.5%) and appendiceal tumors (17.6%). The predominance of colorectal cases likely reflects local referral patterns, regional disease epidemiology, and case selection adopted during the early phase of program implementation. Although the distribution of primary tumors differs from that reported in international registries such as the Spanish REGECOP registry (24), these differences are expected given variations in referral pathways, institutional expertise, and the limited size of this initial cohort.

A proportion of patients presented with recurrent disease (64.7%) and adverse pathological features, including higher tumor grades and frequent lymphovascular invasion. However, most patients have relatively limited peritoneal tumor burdens. The Peritoneal Cancer Index (PCI) remains one of the most important prognostic factors in CRS-HIPEC candidates and plays a significant role in patient selection (26, 27).

Higher PCI scores are consistently associated with lower rates of complete cytoreduction and poorer survival outcomes (28). In our study, 76.5% of patients had low-to-moderate PCI scores, including 56.3% classified as having low PCI scores. The completeness of cytoreduction is the most important surgical prognostic factor in CRS-HIPEC, with CC-0 or CC-1 resection considered the primary operative goal (29). The high rate of complete cytoreduction in our cohort should therefore be interpreted in the context of acceptable PCI values and careful patient selection. Current recommendations emphasize strict selection criteria to maximize therapeutic benefit and avoid laparotomies (25).

CRS-HIPEC remains a technically demanding and time-consuming procedure. In our study, the mean operative time was 430 ± 127 minutes, with an average estimated blood loss of 340 ± 201 mL. These parameters reflect variability related to disease extent, visceral resections, and HIPEC protocols (30). Although postoperative morbidity was common, most complications were low grade, suggesting that the procedure can be delivered with acceptable perioperative safety in carefully selected patients during the early stages of a newly established program. Similar observations have been reported from emerging CRS-HIPEC programs in the region (31, 32).

The strength of evidence supporting CRS-HIPEC varies according to tumor histology. In ovarian cancer, the OVHIPEC randomized trial demonstrated improved survival with HIPEC combined with interval cytoreductive surgery in stage III epithelial ovarian cancer patients (30). In colorectal peritoneal metastases, randomized trials such as PRODIGE-7 and PROPHYLOCHIP have produced mixed results regarding the additional benefit of HIPEC despite the established survival advantage of complete cytoreduction (31, 32). For appendiceal tumors and pseudomyxoma peritonei, registry data consistently demonstrate favorable long-term outcomes following CRS-HIPEC, particularly in patients with low-grade mucinous disease (24, 33). Our study therefore reflects the diverse indications for CRS-HIPEC in contemporary practice.

Regarding the perioperative and oncologic outcomes summarized in **Tables 4** and **5**, postoperative morbidity in our series reflected the complexity of CRS and HIPEC but remained largely manageable. Blood transfusion was required in 47.1% of patients, which may be attributable to the extent of multivisceral resections performed during the early phase of program implementation. Previous studies have reported lower transfusion rates, including 14.8% in the ACS-NSQIP cohort (34). Anastomotic leakage occurred in 5.9% of patients, whereas reoperation was required in 5.9% of patients. Published series have reported leakage rates of approximately 8–10% and reoperation rates ranging from 10.7% to 14% (35, 36). Reoperation was required in 5.9% of European series, whereas it was 10.7–14% in large European series (34).

Given the small sample size and the heterogeneity of published cohorts, these comparisons are intended to provide clinical context rather than direct benchmarking.

Postoperative infections occurred in 29.4% of the patients. Large database studies have reported lower rates of postoperative sepsis and organ–space infection (37). However, variations in patient selection, disease burden, perioperative management, and reporting methods should be considered. Importantly, 91.7% of the complications were Clavien–Dindo grade I–II, indicating that most postoperative events were managed successfully with conservative treatment.

Notably, there was no 30-day mortality in our cohort. Although this finding is encouraging, it should be interpreted cautiously in light of the limited sample size and relatively short follow-up. Overall, our early experience suggests that CRS-HIPEC can be implemented with an acceptable perioperative safety profile in carefully selected patients when supported by multidisciplinary expertise, even during the establishment of a new program in a resource-limited setting.

With respect to survival, the early overall survival (OS) and progression-free survival (PFS) outcomes observed in this study were encouraging. The 12-month OS was 91.7%, whereas tumor recurrence occurred in 35.3% of patients, and the mean progression-free survival was 14.8 months. Previous studies have reported a wide range of survival outcomes depending on the primary tumor type, disease burden, completeness of cytoreduction, HIPEC regimen, and duration of follow-up (38–43). Therefore, comparisons with published series should be interpreted with caution, particularly as our cohort included multiple primary malignancies and represents the initial experience of a newly established CRS-HIPEC program. For patients with pseudomyxoma peritonei, published studies have reported favorable long-term survival following CRS-HIPEC (44), providing additional context for the established role of this treatment in selected disease subtypes.

These findings provide encouraging early evidence that CRS-HIPEC can be delivered safely within a newly established program despite the challenges associated with a resource-limited healthcare setting. Importantly, the principal contribution of this study is not to demonstrate equivalence with established high-volume centers but rather to show that, through careful patient selection, multidisciplinary collaboration, and structured perioperative care, implementation of a CRS-HIPEC program is feasible and may achieve acceptable early clinical outcomes.

Several limitations must be identified. First, the small sample size (n=17) limits generalizability and precludes meaningful subgroup or comparative analyses. Second, the retrospective single-center design has inherent limitations, including possible incomplete data capture and selection bias, particularly because patients underwent rigorous multidisciplinary evaluation and were selected for favorable performance status and potentially resectable disease. Third, the heterogeneity of tumor types and variability of HIPEC protocols may have influenced perioperative and oncologic outcomes. Fourth, no inferential statistical analyses were conducted given the exploratory nature of the study and the limited statistical power. The follow-up duration was relatively short (mean 1.35 years), which limited the assessment of long-term recurrence patterns, disease-free survival and overall survival.

This initial Palestinian experience shows that the implementation of a CRS–HIPEC program in a resource-constrained setting is feasible and can be accomplished with acceptable early postoperative safety and perioperative results with careful multidisciplinary patient selection. These results may serve as a preliminary platform for future prospective and multicenter studies on long-term outcomes, optimization of patient selection criteria, and development of standardized treatment protocols in similar health care settings.

## Conclusion

In this early single-center descriptive experience from a resource-limited setting, implementation of CRS–HIPEC was feasible and associated with acceptable perioperative safety and favorable short-term postoperative outcomes in carefully selected patients. Owing to the small cohort size, heterogeneity of the tumor population and limited follow-up time, the oncologic results should be interpreted with caution and should not be considered evidence of the efficacy of the treatment. Larger prospective, multicenter studies with longer follow-up periods are needed to assess long-term survival outcomes and to optimize patient selection and standardization of therapy.

## Data Availability

The raw data supporting the conclusions of this article will be made available by the authors, without undue reservation.
